# The relationship between serum sex hormone and cardiac echocardiographic findings in healthy men

**DOI:** 10.1038/s41598-022-17101-6

**Published:** 2022-07-27

**Authors:** Yohwan Yeo, Seung Woo Park, Sang-Chol Lee, Yun-Mi Song

**Affiliations:** 1 Department of Family Medicine, Samsung Medical Center, Sungkyunkwan University School of Medicine, Seoul, 06351 Korea; 2 Division of Cardiology, Department of Internal Medicine, Samsung Medical Center, Sungkyunkwan University School of Medicine, Seoul, 06351 Korea

**Keywords:** Cardiovascular diseases, Epidemiology, Hypogonadism, Preventive medicine, Risk factors

## Abstract

Serum sex hormones are known to be associated with cardiovascular disease (CVD), but effects in healthy men on cardiac function have not been well quantified. The authors sought to evaluate an association of sex hormones with cardiac structure and function. Study participants were 857 Korean men without significant cardiovascular diseases participating in the Healthy Twin Study. We estimated the associations of total testosterone (TT) and sex hormone-binding globulin (SHBG) with cardiac structure and function measured by echocardiography using a linear mixed regression model with consideration of random and fixed effects of covariates. The ratio of peak early diastolic velocity of mitral inflow over peak early diastolic mitral annular velocity (E/e’) and left atrial volume index (LAVI), functional parameters reflecting left ventricle (LV) filling pressure, were inversely associated with TT even after further cross-adjustment for SHBG level. There was a positive association between LAVI and SHBG, regardless of TT adjustment. These findings suggest that serum testosterone level is positively associated with LV diastolic function independent of SHBG level.

## Introduction

Low levels of circulating testosterone (T) may be associated with a higher risk of atherosclerosis, coronary artery disease, and cardiovascular events^1–3^. Animal data showed that castration was associated with reduced left ventricular mass (LVM) and reduced ejection fraction^4^. It was also suggested that T replacement therapy might help reduce the risk of morbidity and mortality from heart failure, angina, and myocardial ischemia^3,5–7^. However, prospective observational studies and some interventional studies with T replacement therapy suggest there may be adverse cardiovascular-related events including chest pain, syncope, elevated blood pressure, peripheral edema, and increased ischemic heart diseases in aged men^8–11^.

The associations of T with individual structural and functional components of the heart have been inconsistent between studies. An inverse association of T with LVM^12^, and LV ejection fraction (LVEF)^13^, and a positive association between T and right ventricular mass and volume^14^ were observed. Moreover, studies that focused on the effect of sex steroid therapy for improving cardiac function and structure have also shown inconsistent findings. Some studies reported no effect in healthy young men^15^ and in heart failure patients^16,17^. Another interventional study reported a beneficial effect of T on LV diastolic function, but there was no effect on LV systolic function and LVM^18^.

Sex-hormone-binding globulin (SHBG) modulates the serum free T level. Previous studies have reported independent associations of low SHBG level, after adjusting for TT level, with metabolic risk factors^19^ such as elevation of blood pressure, increase in lipid level, impaired glucose metabolism and atherosclerosis^20^. However, despite these metabolic risks, the relationship between SHBG level and its effect on cardiovascular disease (CVD) has not been studied well. In addition, there is controversy about the relationship of SHBG to CVD and cardiac structure and function function^3,21^.

Given these considerations, we conducted this study to evaluate the associations of T and SHBG with cardiac structure and function measured by echocardiography in men without CVD.

## Methods

### Subjects and study design

The study participants were Korean adult male twins and their family members who underwent echocardiography and measurement of serum T and SHBG level during a health examination for the Healthy Twin Study between July 2009 and January 2015. Details of the methodology of the Healthy Twin Study have been previously reported^22^. In brief, the Healthy Twin Study was a nationwide cohort study recruiting Korean same-sex adult twins (≥ 30 years of age) and their first-degree adult family members from the general population. Among the initial 877 participants, we excluded 20 persons with prostate cancer (n = 2) or previous history of ischemic heart disease (n = 18). Thus, 857 men from 319 families were included in the study, which included 101 pairs of male monozygotic twins. Written informed consent was obtained from all participants. The study protocol was approved by the Institutional Review Board of the Samsung Medical Center (2011-10-025) and was managed in according to the ethical principles of the Declaration of Helsinki.

### Serum concentration of sex-hormone level and covariates

Venous blood sample was drawn after a 12-h overnight fast. Sera were separated immediately by centrifuging and were frozen below − 70 °C. We measured TT and SHBG concentration in a central laboratory immediately after thawing the frozen serum. We measured TT with a chemiluminescence immunoassay using commercial ADVIA Centaur XP kits (Siemens, Erlange, Germany) and SHBG with an electrochemiluminescence immunoassay using MODULAR ANALYTICS E170 analyzer (Roche, Basel, Switzerland). The minimum concentration measurable at the laboratory was 0.35 nmol/ L for TT and SHBG. The inter-assay coefficients of variation were less than 7.6% for TT and 0.35 nmol/ L and less than 2% for SHBG.

Body weight and height were measured twice by trained research assistants according to the standard protocol. We used the average value of the repeated measurements for calculating body mass index (BMI, weight divided by height squared (kg/m^2^)). A trained research assistant measured blood pressure twice using a mercury sphygmomanometer, and we used the averaged value of the repeated measurements for analysis. We measured serum concentrations in fresh sera with commercial kits for glucose by Hexokinase enzymatic assay, high density lipoprotein cholesterol (HDL-C) by enzymatic or homogeneous assay, and triglyceride by enzymatic assay on ADVIA 1650 (Siemens, Germany).

We collected information about pre-existing ischemic heart diseases and cancer, current treatment for hypertension, diabetes mellitus, and dyslipidemia, and health behaviors (smoking habits, alcohol consumption, and physical exercise) using a self-administered questionnaire. We categorized smoking habits into three groups: never smokers, former smokers, and current smokers. Alcohol consumption was categorized into two groups: current drinker and nondrinker. We defined physical exercise by the moderate or higher intensity of regular exercise activity and categorized it into three groups by the frequency of exercise per week (< 1, 1–2, or 3 ≤). We defined hypertension as blood pressure ≥ 140/90 mmHg or taking antihypertensive medication and defined diabetes as fasting serum glucose level ≥ 126 mg/dL or hemoglobin A1C level ≥ 6.5%, or taking a glucose-lowering agent.

### Measurement of echocardiography and covariates

A standard echocardiographic examination following the guidelines of the American Society of Echocardiography^23^ used echocardiography machines (Vivid 7 and Vivid E9, GE Medical System, Horten, Norway, and EKO 7, Medison, Seoul, Korea). All members of the same family underwent echocardiographic measurements taken by a single sonographer with the same machine. Study participants were asked to refrain from smoking, heavy physical activity, and intake of caffeine containing drinks or alcohol for at least 3 h before echocardiographic examination. The following components were measured by M-mode and 2-D echocardiography: End-diastolic left ventricular internal dimension (LVIDd), interventricular septum thickness (IVSd), posterior wall thickness (PWTd), and aortic root diameter. We calculated LVEF using the biplane Simpson’s method and LVM using the Devereux formula (0.8 ×  (1.04 × [ (LVIDd + IVSd + PWTd)^3^–LVIDd^3^]) + 0.6) in grams and indexed LVM to body surface area (g/m^2^) (LVMI)^24^. We measured mitral inflow and annular velocities using conventional Doppler and tissue Doppler imaging (TDI). We measured peak early diastolic velocities (E) and peak late diastolic velocities (A) of mitral inflow were measured using pulsed-wave Doppler at the tip of the mitral valve leaflets. Peak early diastolic mitral annular velocities (e’) and peak late diastolic mitral annular velocities (a’) were acquired on TDI at the septal annulus in an apical 4-chamber view.

We evaluated the reliability of echocardiographic measurements between different machines (GE and Medison) and between different echocardiographers. The inter-machine intra-class correlation coefficients estimated in 21 randomly selected persons were between 0.72 and 0.92 for LV structural and functional measurements. The inter-observer intra-class correlation coefficients in 21 randomly selected subjects ranged between 0.64 and 0.99 for the same echocardiographic measurements.

### Statistical analysis

We examined the distribution of sex hormones and echocardiographic measures by age using linear regression analysis. In order to evaluate the association of TT, and SHBG with structural and functional echocardiographic measures, we estimated β coefficients (95% confidence intervals) by the linear mixed regression model. Prior to this, natural logarithm transformation was done for all echocardiographic measures to improve normality. Then, we calculated percent differences (PD) (95% confidence intervals [CI]) of echocardiographic measures per each standard deviation (SD) increase of age-adjusted sex hormones levels by subtracting 1 from the exponentiated β coefficient. Family and twin information were put in the linear mixed regression model as random effects, assuming that the correlation between observations from related twin or family members was constant regardless of the relationships. Age, hypertension, diabetes, lipid lowering treatment, non-high density lipoprotein cholesterol level, BMI, smoking status, alcohol consumption, and regular physical exercise were put in the model as fixed effects, assuming the levels of these variables did not come from probability distribution. To see the association independent of other hormones, we made additional adjustments for other sex hormones (SHBG for TT and TT for SHBG). All analyses were performed using SAS (version 9.4, Cary, NC). A two-sided p-value < 0.05 was set as statistically significant level.

### Ethics approval and consent to participate

This study was approved by the institutional review board (IRB) of Samsung Medical Center (IRB File No. SMC Center 2011-10-025) and was managed in according to the ethical principles of the Declaration of Helsinki. Informed consent from all individual participants was obtained.

## Results

### Baseline characteristics (Table 1)

**Table 1:** Characteristics of study subjects (N = 857).

Age, years	43.9 ± 14.4
Body mass index, kg/m^2^	24.0 ± 3.2
Hypertension*	185 (21.6)
Systolic blood pressure, mmHg	116.8 ± 16.1
Diastolic blood pressure, mmHg	73.6 ± 10.5
Diabetes mellitus^†^	61 (7.1)
Fasting glucose, mmol/L	5.28 ± 1.09
Lipid lowering medication	32 (3.7)
Total cholesterol, mmol/L	4.87 ± 0.94
High density lipoprotein cholesterol, mmol/L	1.22 ± 0.30
Low density lipoprotein cholesterol, mmol/L	2.89 ± 0.83
Triglycerides, mmol/L	3.56 ± 2.73
Ex-smoker	247 (28.8)
Current smoker	345 (40.3)
Current alcohol drinker	673 (78.5)
**Regular physical exercise**	
1–2/week	157 (18.3)
≥ 3/week	188 (21.9)

Data are presented as mean ± standard deviation for continuous variables or number (%) for categorical variables. *Taking blood pressure lowering medication or having higher blood pressure (≥ 140/or 90 mmHg) †taking antidiabetic medication or having higher level of fasting glucose (≥ 126 mg/dL).

Mean age of the study participants (N = 857) was 43.9 (SD: 14.4) years and mean BMI was 24.2 (SD: 3.2) kg/m^2^. About 21.6% were hypertensives and 7.1% had diabetes mellitus. Prevalence of current smokers was 40.3% and 78.5% were current alcohol drinkers; 21.9% of the study population were doing regular exercise more frequently than twice per week (Table 1).

### Serum sex hormone level and echocardiographic findings according to age (Table 2)

**Table 2:** Distribution of echocardiographic measures by age group.

	Total number of subjects	Overall (N = 857)	< 30 years (N = 125)	30–39 years (N = 270)	40–49 years (N = 155)	50–59 years (N = 157)	60 years ≤ (N = 150)	P for trend*
		Mean (SD)	Mean (SD)	Mean (SD)	Mean (SD)	Mean (SD)	Mean (SD)	
Total testosterone, ng/dL	857	594.0 (215.1)	678.3 (231.7)	581.9 (199.8)	573.0 (216.1)	592.3 (204.0)	568.9 (223.3)	0.0022
Sex hormone binding globulin, nmol/L	856	42.6 (20.1)	30.8 (11.7)	36.3 (15.7)	42.4 (20.0)	48.5 (19.2)	57.6 (23.0)	< 0.0001
**M-mode/2D echocardiography**								
LV mass index (g/ m^2^)	857	76.8 (16.3)	73.2 (14.4)	73.9 (16.1)	76.6 (15.5)	82.0 (15.9)	79.9 (17.7)	< 0.0001
LV internal diameter, diastolic (mm)	857	50.6 (3.7)	51.6 (3.4)	50.7 (3.4)	50.8 (3.6)	50.9 (3.6)	49.0 (3.9)	< 0.0001
LV ejection fraction (%)	857	62.9 (5.7)	61.8 (4.6)	62.2 (5.3)	62.7 (5.6)	64.0 (6.2)	64.1 (6.3)	< 0.0001
Left atrial volume index (ml/m^2^)	852	27.6 (7.2)	26.1 (5.1)	25.5 (5.2)	27.3 (5.2)	29.6 (8.7)	30.6 (9.9)	< 0.0001
Aortic root diameter (mm)	855	31.9 (4.4)	28.8 (3.6)	30.8 (3.3)	32.6 (3.8)	33.4 (4.0)	34.2 (5.4)	< 0.0001
**Doppler echocardiography**								
E/A ratio	845	1.3 (0.6)	2.0 (0.7)	1.5 (0.4)	1.2 (0.4)	1.1 (0.5)	0.8 (0.2)	< 0.0001
E/e’ ratio	843	7.2 (2.3)	6.0 (1.2)	6.5 (1.5)	7.2 (1.8)	8.0 (2.7)	8.7 (2.9)	< 0.0001

*SD* standard deviation, *LV* left ventricle, *E* peak early diastolic velocity of mitral inflow, *A* peak late diastolic velocity of mitral inflow, *e’* peak early diastolic mitral annular velocity.

*Assessed by linear regression analysis.

With the increase of age, the level of TT gradually decreased (p for trend < 0.01), whereas SHBG level gradually increased (p for trend < 0.01). Most echocardiographic parameters representing cardiac structures tended to gradually increase with increasing age, but LVIDd showed an inverse trend. Among functional echocardiographic parameters, E/A ratio (p < 0.0001) showed a decreasing trend, whereas E/e’ ratio increased (p < 0.0001) with age.

### The association between serum sex hormone and cardiac structure and function (Table 3)

**Table 3:** Percent difference (95% confidence intervals)^†^ in echocardiographic measures per 1-SD increase of testosterone and sex hormone binding globulin.

	Percent difference Per 1-SD			
	Total testosterone		Sex hormone binding globulin	
	Model 1	Model 2	Model 1	Model 3
**M-mode/2D echocardiography**				
LV mass index (g/ m^2^)	− 0.6 (− 3.1, 2.1)	− 0.5 (− 3.5, 2.6)	− 0.4 (− 3.3, 2.6)	− 0.1 (− 3.5, 3.4)
LV internal diameter, diastolic (mm)	− 0.6 (− 1.1, − 0.2)	− 0.8 (− 1.4, − 0.2)	− 0.1 (− 0.7, 0.4)	0.3 (− 0.3, 1.0)
LV ejection fraction (%)	− 0.1 (− 0.8, 0.4)	− 0.8 (− 1.5, 0.0)	0.8 (0.1, 1.5)	1.3 (0.4, 2.1)
Left atrial volume index (ml/m^2^)	− 1.0 (− 2.5, 0.5)	− 2.5 (− 4.2, − 0.8)	1.8 (0.1, 3.6)	3.4 (1.4, 5.5)
Aortic root diameter (mm)	− 0.6 (− 1.4, 0.1)	− 0.5 (− 1.4, 0.4)	− 0.6 (− 1.4, 0.3)	− 0.3 (− 1.3, 0.8)
**Doppler echocardiography**				
E/A ratio	− 0.4 (− 2.3, 1.6)	− 0.9 (− 3.2, 1.4)	0.8 (− 1.4, 3.1)	1.4 (− 1.3, 4.0)
E/e’ ratio	− 2.6 (− 4.2, − 1.0)	− 2.5 (− 4.4, − 0.6)	− 1.6 (− 3.4, 0.3)	− 0.1 (− 2.3, 2.1)

*LV* left ventricle, *E* peak early diastolic velocity of mitral inflow, *A* peak late diastolic velocity of mitral inflow, *e’* peak early diastolic mitral annular velocity, *SD* standard deviation † *β* coefficients (95% confidence intervals) for log-transformed echocardiographic measures per 1-SD increase of age-adjusted sex hormones levels were assessed by the linear mixed model. Then, percent differences (95% confidence intervals) in echocardiographic measures were calculated by subtracting 1 from the exponentiated *β* coefficient. Model 1, family and twin were adjusted as random effects, and smoking status, alcohol consumption, regular physical exercise, hypertension, diabetes, lipid lowering treatment, and non- high density lipoprotein cholesterol level were adjusted as fixed effects. Model 2: sex hormone binding globulin was further adjusted in addition to the covariates of model 1. Model 3: total testosterone was further adjusted in addition to the covariates of model 1.

**Figure 1:**
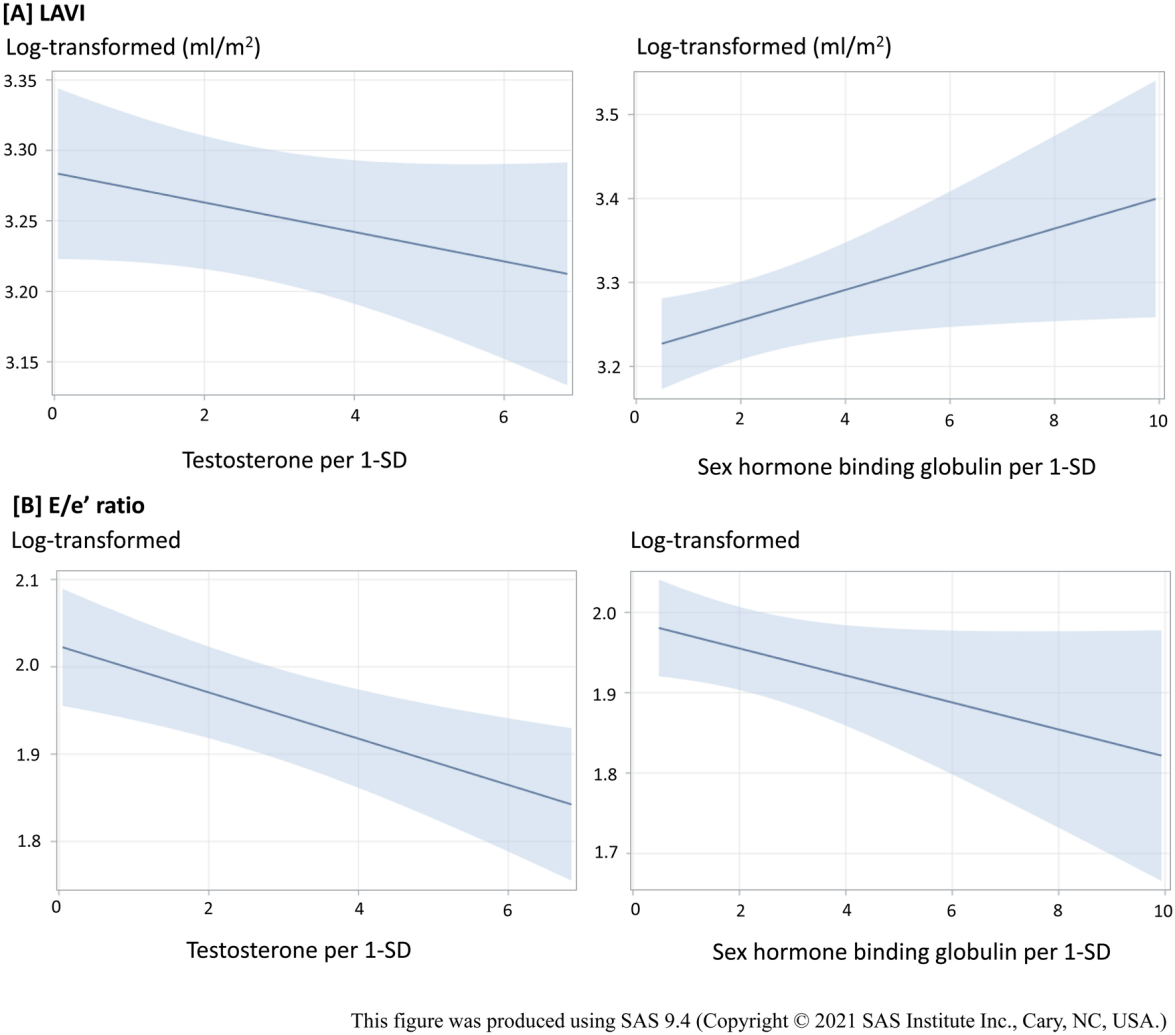
Fitted plots for the association between sex hormone (per 1-standard deviation) and LV diastolic function (log-transformed). *T* tetosterone, *SHBG* sex hormone binding globulin, *LV* left ventricle, *LAVI* left atrial volume index, *E* peak early diastolic velocity of mitral inflow, *A* peak late diastolic velocity of mitral inflow, *e’* peak early diastolic mitral annular velocity, *SD* standard deviation. ^†^Predicted value of log-transformed echocardiographic measures (95% CI) for each sex hormones per 1-SD by the linear mixed model adjusted for family and twin as random effects, and smoking status, alcohol consumption, regular physical exercise, hypertension, diabetes, lipid lowering treatment, and non- high density lipoprotein cholesterol level as fixed effects. SHBG was further adjusted in the analysis of total testosterone and total testosterone was further adjusted in the analysis of SHBG as covariate.

The associations of TT and SHBG with echocardiographic measures were presented as percent differences of echocardiographic measures per a 1-SD increase of age-adjusted levels of each sex hormone. After adjusting for age and cardiovascular risk factors (model 1), TT level was inversely associated with LVIDd and E/e’ ratio. After we further adjusted for SHBG level (model 2), serum TT level was inversely associated with both high E/e’ ratio and LAVI that reflected LV filling pressure. Fitted plots which figured out the association between each sex hormone component and LAVI and E/e’ are also shown in Fig. 1.

The inverse association between TT and LVIDd did not change and the association between TT and LVEF became evident (*p* < 0.05) after we adjusted for SHBG. We found a positive association of SHBG with LAVI and LVEF, regardless of adjusting for TT level (model 3). LVMI did not show any significant association with any of the three sex hormone levels.

## Discussion

In this study of Korean male twins and their male family members, we found that serum TT and SHBG concentration were associated with echocardiographic parameters for LV diastolic function. Decrease of serum TT level was associated with increases in LAVI and E/e’ reflecting LV filling pressure. SHBG level showed a positive association with LAVI, which seemed to suggest a negative role of SHBG for LV diastolic function.

Although the relationship between T and LV diastolic dysfunction has been scarcely studied than has been the relationship between T and parameters for LV systolic function, T has been consistently suggested to play a positive role on LV diastolic function. A study in men with acute heart failure reported that low circulating T level was related to worse LV diastolic dysfunction^25^. Similarly, another study in men with asymptomatic type 2 diabetes^26^ or with type 2 diabetes without structural heart diseases also found that low T was related to the increased LV filling pressure represented by higher E/e’. On the other hand, some studies have suggested T supplementation after myocardial infarction may worsen the patients’ symptoms^27–29^, and Culic et al. postulated that T was beneficial only for relatively normal hearts^30^. Findings of our study which included a large sample of healthy men with no previous history of heart diseases and with relatively well-preserved systolic function (mean LEVF, 62.9%; SD, 5.7%) seems to support the positive role of T on LV diastolic function in healthy men.

The mechanism underlying the association between T and LV diastolic function has not been clearly understood yet. As a possible explanation, the inhibition of cardiac fibroblast migration and proliferation and myofibroblast differentiation mediated by growth factor-β in the presence of T has been suggested by an in-vivo study^31^. Also, the anti-fibrotic effect of T might play a protective role by attenuating oxidative stress in rats model without myocardial infarction^30^. However, exogenous T given to rats with cardiac diseases including myocardial infarction worsened cardiac dysfunction, negating the favorable effect by T in unhealthy heart^27,30^.

Worsening of vasoconstriction, inflammation, and atherosclerosis associated with decrease in serum T level may explain the increased metabolic and CVD risk in men with low T level^2^, although our study did not address this issue. In a vivo study, T was found to reduce vascular resistance and to increase arterial blood flow^32^. In vivo studies suggested that T may increase and promote proliferation of vascular smooth-muscle cells^33,34^.

There have been studies supporting the beneficial role of T on congestive heart failure. It is reported that there are sex hormone receptors in the human myocardial cells^35,36^. Men with moderately severe heart failure were found to have T deficiency^6,37^ and T replacement in men with T deficiency has improved functional capacity, performance, and quality of life of patients probably through an anabolic effect on skeletal muscle^17,38^. In some clinical trials, T replacement in androgen-deficient men with congestive heart failure has improved functional and exercise capacity^5,39^.

However, the role of T on systolic cardiac function in healthy men has been inconsistent. A cross-sectional study of 1223 middle-aged healthy men reported that T was inversely associated with LVEF^34^. Among athletes, short-term administration of anabolic steroids did not result in significant alteration of LV structure or LVEF^40^. Some clinical and preclinical models suggested that a supraphysiologic high level of T might be detrimental to the myocardium^15,21^. Animal studies also suggested that endogenous sex-hormones within a physiologic range of concentration were less likely to control cardiac contractility, although the findings were inconsistent in clinical trials^4,41,42^.

In our study, we found no significant association between sex hormones and LVMI after adjusting covariates including BMI. It has been suggested that endogenous T level influences LVM formation, because LVM does not change in men with advancing age but tends to increase in women^43,44^. Although the mechanism underlying the relationship between T and cardiac mass needs to be further clarified, in vivo studies suggested that T may increase and promote myocardial inflammatory response, and cardiac remodeling, and thus, increase LVM^45,46^. However, epidemiological studies have reported controversial findings. In the study of 1264 healthy men aged between 25 and 84 years, TT was inversely associated with LVM and men with LV hypertrophy had significantly lower levels of TT, although this association did not persist after adjusting for BMI^12^. Another study of healthy middle-aged men found no independent association between T and LVM^47^. Therefore, the relationship between sex-hormones and cardiac structure need to be further investigated.

The relationship of SHBG with cardiac structure and function has hardly been evaluated. In this study, the association of SHBG with echocardiographic parameters reflecting diastolic filling dysfunction was inconsistent: a positive association with LAVI and null association with E/e’. In our study, SHBG was positively associated with LVEF, a parameter of systolic function, suggesting that SHBG affect cardiac systolic function in an opposite direction to T. However, this finding differs from the finding of a study in male patients with chronic heart failure that SHBG level was inversely associated with LVM and LVEF. Our study finding is also different from the finding of a community study that SHBG was inversely associated with LVM but not with LVEF^48^. Thus, the relationship of SHBG to cardiac structure and function seems still unclear.

There are some limitations in this study. First, this study is a cross-sectional observational study and we evaluated the association of single measurement of sex hormone levels and echocardiographic parameters. Therefore, any causality or direction of the observed association could not be clearly proved. Second, several echocardiographers did echocardiographic measurement using two different types of machines, which might have resulted in measurement bias. However, given that intra-class correlations for assessing inter-observer and inter-machine repeatability were moderate to high, significant bias was less likely. Third, we measured T concentration using a radioimmunoassay. Although mass spectrometry is more accurate and is the gold standard, it is more time-consuming and expensive than is radioimmunoassay. Fourth, we could not evaluate the association between bioavailable T and echocardiographic parameters due to the lack of directly measured bioavailable T data. Free T level is known to be estimated by Vermeulen’s method using the measured levels of TT and SHBG^49^. However, we did not include the findings related to the calculated free T level in the final analysis, because there could be a concern that calculated free T does not accurately reflect bioavailable T level^50,51^. Fifth, we did hormone measurements only once. At last, residual confounding effects by unmeasured genetic factors could be possible. In our previous studies using the data from the Healthy twin Study, cardiac structure^52^ and sex steroids^53^ were found to have low to moderate level of heritability. Although common genetic factors shared between sex hormones and cardiac measures were not identified yet, pleiotropic effect through shared gene on the observed phenotypic association could not be excluded.

This study also has some strengths. Our study was a community-based study that included healthy men without a previous history of heart diseases or androgen deprivation therapy, which allowed us to examine the association between sex steroids and cardiac measures within a physiologic range. In addition, although clinical significance is still uncertain, we evaluated the associations of SHBG with cardiac measures.

## Conclusions

Our study in healthy Korean men revealed that TT were inversely associated with echocardiographic parameters reflecting LV filling pressure such as LAVI and E/e’. These findings suggest that serum testosterone level is positively associated with LV diastolic function.

## Data Availability

The datasets used for this study are available from the corresponding author on reasonable request.

## Abbreviations

T: Testosterone
TT: Total testosterone
SHBG: Sex hormone-binding globulin
LVM: Left ventricular mass
LVIDd: Diastolic left ventricular dimension
LAVI: Left atrial volume index
LVEF: Left ventricular ejection fraction
E: Peak early diastolic velocity of mitral inflow
A: Peak late diastolic velocity of mitral inflow
e’ peak: Early diastolic mitral annular velocity
a’ peak: Peak late diastolic mitral annular velocity
